# Prevention of mother-to-child transmission of HIV in Kermanshah, west of Iran from 2014 to 2021

**DOI:** 10.1186/s12887-022-03829-7

**Published:** 2023-01-19

**Authors:** Roya Chegene Lorestani, Mosayeb Rostamian, Alisha Akya, Shahab Rezaeian, Mandana Afsharian, Reza Habibi, Arezoo Bozorgomid, Narges Kazemisafa, Somayeh Jafari, Soliman Yeilaghi, Mansour Mohammad Salehi, Hiva Namdari, Keyghobad Ghadiri

**Affiliations:** 1 Infectious Diseases Research Center, Health Institute, Kermanshah University of Medical Sciences, Kermanshah, Iran; 2 Clinical Research Development Center, Dr. Kermanshahi Hospital, Kermanshah University of Medical Sciences, Kermanshah, Iran; 3 School of Medicine, Kermanshah University of Medical Sciences, Kermanshah, Iran; 4 Clinical Research Development Center, Imam Reza Hospital, Kermanshah University of Medical Sciences, Kermanshah, Iran; 5 Expert Advice Center for Behavioral Diseases, Kermanshah University of Medical Sciences, Kermanshah, Iran

**Keywords:** Effectiveness, HIV, Prevention of mother-to-child transmission (PMTCT)

## Abstract

**Background:**

This study aimed to evaluate the implementation of the prevention of mother-to-child transmission (PMTCT) of the HIV-PMTCT program in Kermanshah, west of Iran, from 2014 to 2021.

**Methods:**

The data of all HIV-infected mothers and their infants who were monitored by the Kermanshah behavioral diseases counseling center was extracted and recorded in a checklist.

**Results:**

Out of 95 included infant, 45 (47.4%) were girls and 50 (52.6%) were boys. The mothers were mostly infected with HIV via their infected spouse. The pregnancies of 77 cases (82.1%) were in accordance with the national guideline. The average length of treatment for this group was 185 days. Of the 18 mothers who did not receive treatment, nine were diagnosed during childbirth and nine had no available information. All infants born from infected mothers underwent after-birth-antiretroviral prophylaxis, and all remained healthy. There was no statistically significant relationship between the birth weight and height of neonates with maternal age, maternal last viral load, disease stage, education, and maternal CD4 levels. Only a statistically significant relationship was observed between the duration of treatment and the infants’ weight.

**Conclusion:**

The results suggest the feasibility and effectiveness of the PMTCT program for HIV-positive mothers in Kermanshah. It seems that if pregnant HIV-positive women are diagnosed early and covered by a good prevention program on time, the risk of HIV to their babies will be reduced, significantly.

## Background

Research indicates 38 million people lived with HIV worldwide by 2020, and 2.78 million of them were children and teenagers. Approximately 850 children are infected with HIV every day, and most of them become infected through mother-to-child transmission MTCT [1]. Maintaining the health of pregnant mothers and the plan to eliminate HIV-MTCT are among the priorities of the World Health Organization (WHO) [2], as a global guide to eliminate HIV-MTCT published in 2014 [3].

In the Middle East, Iran was a pioneer in the implementation of the PMTCT [4]. However, the number of people living with HIV/AIDS in the country is still high, and it has recently been reported that the mode of HIV transmission has been changing from drug injections to sexual contact [5]. In recent years, the number of HIV-infected women of childbearing age has increased, which in turn increases the transmission of the virus from mother to child [6]. MTCT occurs during pregnancy, childbirth, or breastfeeding [7, 8]. According to the Iranian Ministry of Health, the number of HIV-infected babies born from infected mothers is increasing. Therefore, great attention should be paid to HIV-infected mothers and their infants as the principal part of the HIV/AIDS control program [9]. The PMTCT program includes the prevention of infection in young women, the prevention of unwanted pregnancies, care of mothers during pregnancy, childbirth, and breastfeeding, as well as the care of infants born from HIV-infected mothers [10].

One of the great achievements of global public health over the last 20 years is the implementation of the PMTCT program. The MTCT rate is 15–25% in developed countries and 25–45% in developing countries without prevention programs [11]. If the PMTCT program is used, the MTCT rate will go down less than 1% in developed countries and slightly higher in developing countries [12–15].

The lack of proper service and commitment to PMTCT are two main obstacles to eliminating HIV-MTCT [16, 17]. Reasons such as stigma, disclosure, lack of access to antiretroviral therapy (ART), poor awareness of MTCT or PMTCT programs, and the lack of male involvement lead to incomplete adherence to PMTC procedures [18–24]. Therefore, early diagnosis of HIV-infected mothers and applying proper interventions to increase their adherence to the PMTCT program is crucial. Since 2014, the PMTCT program was implemented in Iran, including Kermanshah province. This study aimed to evaluate the implementation of the PMTCT of HIV-PMTCT program in Kermanshah from 2014 to 2021.

## Methods

This study was performed on HIV/AIDS patients referred to and monitored by the Kermanshah behavioral diseases counseling center (Affiliated with the Kermanshah University of Medical Sciences) from 2014 to 2021.

Pregnant women referred to the counseling center are screened routinely, and if HIV infection is confirmed in them, drug prophylaxis is started. Preparatory work for cesarean delivery, coordination for the preparation of powdered milk, and care related to the baby are provided free of charge and with confidentiality at the counseling center. All information about the mother and the infants is recorded in proper files.

The data of mothers and their infants were recorded regarding associated variables. These variables included maternal delivery method, the way of mother infection, prophylactic drugs used for infants, congenital anomalies at birth, infant weight at birth, maternal and neonatal viral load based on real-time PCR, maternal CD4 count, gestational age (years), diagnosis time in mother relative to birth time (months) and duration of maternal treatment during pregnancy (days).

Neonates born from HIV-infected mothers were monitored up to two years old, and their growth indices were assessed. Consequently, the data from those monitored by the program for two years were collected from their files. All high-risk infants were included, as soon as they were born and the HIV pediatric specialists for HIV focal unit were notified. At the counseling center, patients were examined for viral load at birth, at two months, and 4–6 months later by real-time PCR, and after 18 months after childbirth by ELISA methods. For this study, the ethics approval was taken from the Ethics Committee of Kermanshah University of Medical Sciences (IR.KUMS.REC.1398.137).

The data analysis was performed using STATA 14 software at a 95% confidence interval. Mean standard deviation, minimum and maximum were reported for continuous variables. Qualitative variables were presented by frequency and percentage. The normality of data was checked by the Kolmogorov–Smirnov test. The correlation coefficient, independent t-test, and one-way ANOVA were used to determine the association between variables.

## Results

Of 95 included infant, 45 (47.4%) were girls, and 50 (52.6%) were boys. The average weight, height, and head circumference were 3010.66 ± 600.81 g, 50.17 ± 3.10 cm, and 34.80 ± 1.56 cm, respectively.

Demographic information of the mothers is presented in Tables 1 and 2. Most mothers were aware of their prenatal status of HIV infection, so the mean time from the infection diagnosis until birth was 32.69 ± 35.09 months. Most infants (84 cases, 88.4%) were born by cesarean section.

**Table 1:** Information of the mothers

Variable	Mean	Min	Max
Diagnosis time in mother relative to birth time (months)	32.69 ± 35.09	1	149
Duration of maternal treatment during gestational (days)	185.15 ± 77.2	30	270
Last CD4	496.95 ± 289.53	83	1434
Mother's age (years)	35.9 ± 5.5	24	47
Gestational age (years)	29.80 ± 5.39	20	42
Mother's latest viral load	45.10 ± 112.22	0	408

**Table 2:** Parental demographic information in mother

Parameters		Frequency (%)
Maternal treatment during gestational	Yes	77(81.1)
	No	9(9.4)
	Incomplete treatment	3(3.1)
	Unknown	6(6.3)
Transmission routes	Sex / Injection	3(3.1)
	Sex	11(11.5)
	Spouse of the infected person	75(78.9)
	Unknown	6(6.3)
Disease stage	Stage I	6(6.3)
	Stage II	71(74.7)
	Stage III	3(3.1)
	Stage IV	2(2.1)
	Unknown	13(13.6)
Previous marriage	Yes	25(26.8)
	No	60(64.5)
	Unknown	8(8.6)
Education	Illiterate	14(14.7)
	Elementary	36(37.8)
	Elementary/Middle school	25(26.3)
	University	2(2.1)
	Unknown	18(18.9)
Job	Housekeeper	84(88.4)
	Employed	2(2.1)
	Unknown	9(9.4)
Addiction	Yes	10(10.5)
	No	82(86.3)
	Unknown	3(3.1)
Method of delivery	Cesarean	84(88.4)
	Natural	7(7.3)
	Unknown	4(4.2)
Receiving zidovudine during delivery	Yes	60(63.1)
	No	23(24.2)
	Unknown	12(12.6)
Prison history	Yes	7(7.3)
	No	83(87.3)
	Unknown	5(5.2)
Sex with others	Yes	13(13.6)
	No	74(77.9)
	Unknown	8(8.4)

Seventy-seven (82.1%) pregnant women underwent antiretroviral treatment. The mean duration of treatment in mothers during pregnancy was 185.15 ± 17.2 185 days. Antiviral treatment was not prescribed for nine (9.4%) mothers because they were diagnosed during delivery. Six (6.3%) infants were from orphanages, and it was not known whether their mothers were treated during pregnancy or not. In three (3.1%) other children from orphanages, the mother had not received regular treatments.

The viral load with a mean of 45.10 ± 112.22 (copies/ml) was reported in 24 cases of prenatal mothers. All infants received prophylaxis according to the PMTCT protocol and were treated with antiretroviral drugs for 42 days. Seventy-two cases (75.8%) were treated with cotrimoxazole from 6 weeks of age.

Regarding the infection of children’s fathers, 27% were infected by injection, 21.1% by sex, and 20% by sex/injection, 1.1% were spouses of the infected person, 14.7% not infected, and 15.7% were unknown. However, all included mothers were HIV infected, and 78.9% were infected through the infected-spouse, 11.5% through sex, 3.1% through sex/injection, and 6.3% were unknown.

Analysis of the relationship between neonates’ height, weight, and head circumference at birth with mothers’ factors such as the duration of treatment, the last viral load, age, and the time of diagnosis relative to the time of birth, showed no statistical significant association, except the relationship between the duration of maternal treatment with infants’ birth weight (*p* = 0.001) (Table 3).

**Table 3:** The relationship between height, weight, and head circumference at birth with the duration of treatment, last viral load, and age in the mother

Mothers’ characteristics	Neonates characteristics	Spearman's rho	*p*-value
Length of treatment of the mother	Birth weight	0.5384	0.001
	Birth height	0.268	0.2279
	Birth Head Circumference	0.0957	0.7055
Viral loaded mother	Birth weight	0.3754	0.168
	Birth height	0.1519	0.6043
	Birth Head Circumference	0.023	0.9465
Maternal age	Birth weight	0.732	0.5916
	Birth height	-0.0536	0.7948
	Birth Head Circumference	-0.2885	0.2046
Diagnosis time in the mother relative to the time of birth	Birth weight	-0.1071	0.4454
	Birth height	0.4567	0.019
	Birth Head Circumference	0.1049	0.6423

In the treated mothers, the height, weight, and head circumference of neonates at birth were higher than those of the untreated mothers, but the difference was not statistically significant. The mean duration of treatment was longer in mothers with a CD4 above 200 (cell/mm3) than in those with a CD4 below 200 (cell/mm3), but it was not statistically significant. There was no relationship between the mother's education level and the mother's disease stage with infants' birth weight (Table 4).

**Table 4:** The relationship between height, weight, and head circumference at birth with disease stage, education, CD4, and treatment in the mother

			**means of parameters**	**Std. Dev**			***p*-value**
**Birth weight**	**Disease stage**	1	3700	0	Test of Heterogeneity		
		2	3007.8	62,317,584	231,241.3		0.647
		3	2900	0			
		4	3350	70,710,678			
		Unknown	2825	51,744,565			
**Birth weight**	**Education**	Illiterate	3410	33.24	390,705.754		0.298
		Preliminary	2972.27	511.3			
		Higher	3045	643.57			
**Birth height**	**Education**	Illiterate	50.33	2.081	9.14393939		0.395
		Preliminary	51.5	3.65			
		Higher	49.6	2.61			
**Birth Head Circumference**	**Education**	Illiterate	34.5	1.32	3.415625		0.31
		Preliminary	34.3	1.5			
		Higher	35.5	1.87			
**Length of treatment of the mother**	**CD4**	< 200	167.16	78.32	% 95conf. Interval		0.665
					84.97	249.35	
		≥ 200	181.03	73.5	160.97	201.1	
**Birth weight**	**CD4**	< 200	2870	405.58	2366.39	3373.6	0.535
		≥ 200	3058.7	655.59	2846.19	3271.23	
**Birth weight**	**Maternal treatment**	Yes	3032.5	611.12	2862.36	3203.63	0.477
		No	2868.7	543.09	2414.7	3322.79	
**Birth height**	**Maternal treatment**	Yes	50.52	2.92	49.25	51.78	0.216
		No	48.6	3.78	43.9	53.29	
**Birth Head Circumference**	**Maternal treatment**	Yes	35.02	1.47	34.29	35.76	0.201
		No	34	1.76	31.8	36.19	

The correlation between the mother's last CD4 and gestational age was -0.06 and was not significant (*p* = 0.0614). Also, the correlation between the last viral load of the mother and the gestational age was not significant (*p* = 0.14). There was no correlation between gestational age and birth weight (*p* = 0.21). Only the positive correlation between the mother's last CD4 and birth weight was significant; in other words, with the increase of the mother's CD4, the birth weight also increased (*p* = 0.02).

PCR was performed at about 48 after birth to 6 weeks (mean 25.54 ± 14.2 days after birth) and from 2 to 4 months (mean 76.5 ± 23.2 days after birth) in 67 cases (70.5%). The results of both PCR tests were negative in all of them.

One of the infants (1.05%) had only one negative PCR test, which is related to before the PMTCT program in Kermanshah.

At the beginning of this program in Kermanshah, PCR test was not easily available in Kermanshah behavioral diseases counseling center, so in 17 infants (17.8%) only ELISA and in 10 infants (10.5%) ELISA and PCR were performed. The results of PCR and ELISA were negative in all infants.

## Discussion

In recent years, the number of women living with HIV in Iran has increased, so more than 16% of people living with HIV in Iran are women. Most infected women are of childbearing age, and if they become pregnant, they may transmit the virus to their children [25].

In a retrospective study that we conducted on HIV/AIDS patients referred to the Kermanshah behavioral diseases counseling center between 2011 and 2019, the results showed that the number of women with HIV has increased, so that the percentage of women with HIV from 24.3% in 2011 reached 41.2% in 2019 [26]. In a study in Kermanshah during the years 1996–2014, the percentage of women living with HIV increased as in the years 1996–2002, out of 665 HIV-infected people, 6 cases (0.9%) and during the years 2009–2014, out of 955 HIV-infected people, 146 cases (15.2%) were women [27]. This indicates the requirement for preventive treatment for all HIV-infected women who wish to become pregnant.

In Iran, the use of antiretroviral drugs to prevent MTCT of HIV was established in 2006 [28]. It has been reported that due to the expansion of the PMTCT program in most parts of Iran, the rate of HIV-MTCT was decreased, but due to the increase in the number of women living with HIV, the number of infected infants is relatively constant [29]. Nevertheless, with the implementation of this program in Kermanshah, none of the newborns whose mothers entered the PMTCT program were infected with HIV. Similarly, in a study in Tehran (the capital city of Iran) during 2014–2017, all newborns of 54 infected mothers who entered the PMTCT program were healthy [30]. Likewise, in another study in Iran, only one HIV-infected newborn (1.5%) from mothers who followed the PMTCT program was detected [31]. These results indicate the effectiveness of the PMTCT program in preventing HIV in neonates.

The rate of MTCT in Iran from 1967–2018 was reported 1.6% by Seyedalinaghi et al. [29]. In China, due to investments in the implementation of the PMTCT program, the rate of vertical transmission has decreased [32, 33], from 31.8% before the start of PMTCT to 2.3% after it (in 2011) [34]. Another study evaluating the PMTCT program in China reported that the rate of MTCT of HIV decreased from 19.4% in 2010 to 9.6% in 2016 [35]. Although good progress has been made in China, there is still a long way to go to reduce transfers to zero [36]. HIV-MTCT has been eliminated in Cuba, Belarus, and Thailand [37]. In the UK, the MTCT rate has dropped to less than 1% [38]. Factors influencing MTCT success include the lack of treatment during pregnancy, normal delivery, failure to receive postpartum treatment, and failure to treat prophylaxis in infants.

Research in Iran has reported that treatment with antiretroviral drugs increased by 55.5% in CD4 counts and decreased by 84.8% in viral load [31]. In our study, the CD4 level of most mothers (71%) treated during pregnancy was higher than 200 (cell/mm3). However, there was no significant relationship between the duration of maternal treatment and the amount of CD4; because only six pregnant mothers had a CD4 lower than 200 (cell/mm3). Moreover, in our study, the viral load data was only available for 24 cases (25%) of mothers before delivery, in 18(75%) of them, the viral load was zero.

One of the important factors that prevent the full implementation of the PMTCT program is that several mothers do not have any check-ups for HIV/AIDS before getting pregnant or seek treatment late in pregnancy [39–41]. Some researchers believe that antiretroviral drugs are harmful to the fetus and disagree with the use of antiretroviral drugs. Despite these oppositions, prenatal screening and the use of antiretroviral drugs should be expanded because of the benefits to mother and child health [42]. In our study, 77 (82.1%) of pregnant women were covered by the treatment guideline for HIV-positive women. The mean duration of treatment in mothers during pregnancy was 185.15 ± 17.2 days. Mothers diagnosed during childbirth were treated during and after delivery, and all babies born to HIV-positive mothers underwent prophylaxis. In a study from Tehran, out of 15 pregnant mothers who did not enter the PMTCT program, three (20%) neonates were infected with HIV [30].

Research suggests that cesarean section reduces mother-to-child transmission of HIV, but its surgical complications and economic burden for mothers are high [28, 43–45]. The WHO does not recommend an elective cesarean section [46, 47], but the late onset of antiretroviral prevention during pregnancy may justify the high rate of cesarean section for PMTCT [34]. As reported by another study from Iran, since viral load and CD4 testing are performed only for a small number of cases, cesarean delivery is recommended for all patients [31].

In our study, 89.2% of mothers had cesarean sections, and only a few percent had a normal delivery. Similarly, in another study in Iran, 5.4% of mothers had a normal delivery, but all of them were diagnosed as HIV-infected cases at the time of delivery [31]. In another report, HIV infection was diagnosed in 56% of mothers before the delivery time [48].

To reduce the number of children infected with HIV, it is necessary to perform PMTCT intervention and provide antiretroviral treatment during pregnancy, childbirth, breastfeeding, and in newborns [49]. In our study, most mothers (85.2%) were aware of their infection status before delivery. It has been reported that in case of non-intervention, the HIV-MTCT rate will be 15–45% [50]. A study by Sakha et al. indicated that the intervention was effective in the awareness of mothers at risk [51]. Due to the cultural problems in Iran, the rate of counseling of HIV/AIDS patients is low, and some even refuse to perform HIV testing [31]. However, the availability of counseling services to inform mothers, the presence of knowledgeable staff, as well as the mothers' higher level of education for better acceptance of antiretroviral drugs, cesarean section, and not using breastfeeding can reduce MTCT to less than 2% [52–54].

The mothers who used antiretroviral drugs, in addition to preventing the transmission of the virus, also reduced the low weight of birth [55]. In our study, a significant relationship was observed between the duration of maternal treatment during pregnancy and the weight of the infant, so the longer the mother's treatment, the higher the neonate's weight at birth. Also, the weight was higher in infants from treated mothers compared to those who were not treated at all, although it was not statistically significant.

In this study, the neonates' mean weight, height, and head circumferences at birth were in the normal range. Also, a study on the European population reported that the mean weight and height of healthy infants born to HIV-infected mothers were 3900 and 52.65, respectively [56]. These values are similar to those reported for infants from non-infected mothers by CDC (3250, 49.51, and 34.10 for weight, height, and head circumference, respectively) [57]. In contrast, some studies have reported that non-infected children at risk of infection have poorer growth than children who were not at risk [58–60]. Reports have shown that the growth of non-infected but at-risk children (from their HIV-infected mothers) is not affected by the virus [61–64]. However, the health and education status of HIV-infected mothers, the economic and social status of the family, and nutritional and health support [65, 66] are the factors that affect the growth of the baby [67, 68]. For instance, a study in South Africa reported that children born to HIV-infected mothers weighed less than international standards, possibly due to environmental and economic conditions [69].

Antiretroviral prophylaxis is recommended in the first six weeks of a baby's life to reduce the risk of vertical transmission [70]. In Iran and the city of Kermanshah, the PMTCT program for HIV infection is being implemented. In the present study, all infants born from HIV-infected mothers underwent prophylaxis by receiving zidovudine from birth to 42 days and also by receiving lamivudine and nevirapine in some infants. Most of the infants received cotrimoxazole from 42 days to 6 months. Overall, in the present study, none of the infants of mothers who entered the PMTCT program became infected with HIV, which is a great success. The main reason for the program's success is access to pediatric HIV focal points, HIV focal points physicians, and staff of the behavioral disease center for the PMTCT program 24 –hours.

The limitations of our study include: 1- unavailability of viral load and CD4 information for some of the mothers 2- Not performing PCR tests for some of the infants 3- Identifying some mothers during childbirth, hence not receiving antiretroviral drugs during pregnancy 4- The residence of some infants in nurseries and lack of information about their parents.

## Conclusion

Our results demonstrated the feasibility and effectiveness of the PMTCT program for HIV-positive mothers in our region. Considering the increasing number of HIV-infected women at childbearing age in Kermanshah in recent years, it is important to focus on PMTCT implementation with early diagnosis and make it more accessible.

## Data Availability

All the data supporting the findings are contained within the manuscript.

## Abbreviations

PMTCT: Prevention of mother-to-child transmission
WHO: World Health Organization
MTCT: Mother-to-child transmission
ART: Antiretroviral therapy
